# Transcriptome Changes in Three Brain Regions during Chronic Lithium Administration in the Rat Models of Mania and Depression

**DOI:** 10.3390/ijms22031148

**Published:** 2021-01-24

**Authors:** Dawid Szczepankiewicz, Piotr Celichowski, Paweł A. Kołodziejski, Ewa Pruszyńska-Oszmałek, Maciej Sassek, Przemysław Zakowicz, Ewa Banach, Wojciech Langwiński, Kosma Sakrajda, Joanna Nowakowska, Magdalena Socha, Ewelina Bukowska-Olech, Joanna Pawlak, Joanna Twarowska-Hauser, Leszek Nogowski, Janusz K. Rybakowski, Aleksandra Szczepankiewicz

**Affiliations:** 1 Department of Animal Physiology, Biochemistry and Biostructure, Poznan University of Life Sciences, 60-637 Poznan, Poland; pawel.kolodziejski@up.poznan.pl (P.A.K.); ewaprusz27@gmail.com (E.P.-O.); maciej.sassek@up.poznan.pl (M.S.); leszek.nogowski@up.poznan.pl (L.N.); 2 Department of Histology and Embryology, Poznan University of Medical Sciences, 60-781 Poznan, Poland; p.celichowski@gmail.com; 3 Department of Psychiatric Genetics, Poznan University of Medical Sciences, 60-806 Poznan, Poland; przemek@zakowicz.eu (P.Z.); joanna.pawlak@gmail.com (J.P.); joanna.hauser@gmail.com (J.T.-H.); 4 Laboratory of Neurobiology, Department of Molecular and Cellular Neurobiology, Nencki Institute, 02-093 Warsaw, Poland; ewabanach01@gmail.com; 5 Molecular and Cell Biology Unit, Poznan University of Medical Sciences, 60-572 Poznan, Poland; wlangwinski654@gmail.com (W.L.); kosma.sakrajda@gmail.com (K.S.); asianowakowska781@gmail.com (J.N.); 6 Department of Medical Genetics, Poznan University of Medical Sciences, 60-806 Poznan, Poland; msocha@ump.edu.pl (M.S.); ewe.olech@gmail.com (E.B.-O.); 7 Department of Adult Psychiatry, Poznan University of Medical Sciences, 60-572 Poznan, Poland; janusz.rybakowski@gmail.com

**Keywords:** manic-like behavior, depressive-like behavior, lithium, animal model, transcriptome, brain

## Abstract

Lithium has been the most important mood stabilizer used for the treatment of bipolar disorder and prophylaxis of manic and depressive episodes. Despite long use in clinical practice, the exact molecular mechanisms of lithium are still not well identified. Previous experimental studies produced inconsistent results due to different duration of lithium treatment and using animals without manic-like or depressive-like symptoms. Therefore, we aimed to analyze the gene expression profile in three brain regions (amygdala, frontal cortex and hippocampus) in the rat model of mania and depression during chronic lithium administration (2 and 4 weeks). Behavioral changes were verified by the forced swim test, open field test and elevated maze test. After the experiment, nucleic acid was extracted from the frontal cortex, hippocampus and amygdala. Gene expression profile was done using SurePrint G3 Rat Gene Expression whole transcriptome microarrays. Data were analyzed using Gene Spring 14.9 software. We found that chronic lithium treatment significantly influenced gene expression profile in both mania and depression models. In manic rats, chronic lithium treatment significantly influenced the expression of the genes enriched in olfactory and taste transduction pathway and long non-coding RNAs in all three brain regions. We report here for the first time that genes regulating olfactory and taste receptor pathways and long non-coding RNAs may be targeted by chronic lithium treatment in the animal model of mania.

## 1. Introduction

Bipolar disorder (BD) is a severe, recurrent psychiatric condition characterized by episodes of mania or hypomania and depression. Mania presents with hyperactivity, disinhibited behavior and inflated self-esteem. On the other hand, during depression patients demonstrate anhedonia and loss of motivation and interest in usual activities [1].

Pharmacological treatment of BD includes mostly the use of mood stabilizers. The first drug fulfilling criteria for the mood stabilizer such as preventing manic and depressive episodes was lithium. Its antimanic actions in the acute episode were first discovered by Cade et al. in animal model studies [2]. Its antidepressant potential was reported in the 1970s [3,4] and later it was recommended as an augmentation of antidepressants in the treatment-resistant depression and an effective long-term prophylaxis of recurrences in mood disorders (as recently reviewed by Rybakowski [5]).

Although lithium has been for decades in clinical use, the molecular mechanism of action and normotymic potential of lithium are still not well identified. Moreover, absence of suitable animal model of bipolar disorder impedes studies on lithium action. Previous studies focused on identifying target genes and molecular pathways of lithium indicated its role in reducing inositol signaling [6], phosphorylation of AKT [7] and inhibition of GSK-3β (glycogen synthase kinase 3) signaling [6,8]. The latter two may lead to the changes in the activation of WNT pathway. These findings were possible to discover using pharmacological and pharmacogenetic studies, which showed involvement of lithium in multiple cellular signaling pathways as reviewed previously [9,10].

Animal studies of the brain transcriptome during lithium administration showed that inconsistent results are mainly due to the different duration of lithium administration (1 week, two weeks and 6 weeks), analysis of the whole brain tissue (whole brain homogenates) and the use of different animals (rat or mouse) [11,12]. Moreover, these studies used animals treated with lithium, but without manic-like or depressive-like symptoms, so it is still not known if therapeutic action of lithium in manic and depressive behavior induces changes in the gene expression of similar or different pathways depending on the baseline condition and the brain region.

Therefore, we hypothesized that chronic lithium treatment exerts its therapeutic effect by inducing changes in the brain transcriptome specific for mania and depression. The aim of this study was to investigate if chronic lithium administration in rats presenting manic-like behavior (induced by amphetamine) or depressive like behavior (induced by chronic mild stress) influences specific gene expression profiles in different brain regions (amygdala, frontal cortex and hippocampus).

## 2. Results

### 2.1. Behavioral Changes in Amphetamine-Exposed and Stress-Exposed Animals (Models of Mania and Depression)

**Figure 1:**
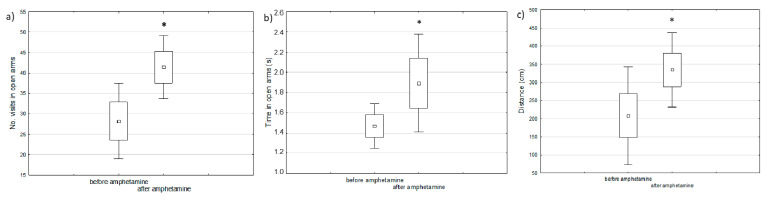
The comparison of behavioral changes before and after amphetamine injection in the elevated maze test: (**a**) number of visits in open arms, (**b**) time spent in open arms, (**c**) distance passed by the animals (paired *t*-test, * defines *p* < 0.05).

Manic-like behavior was observed after one week of amphetamine injections using the elevated maze test as compared to the behavior of the same animals before starting the experiment (baseline) (Figure 1).

**Figure 2:**
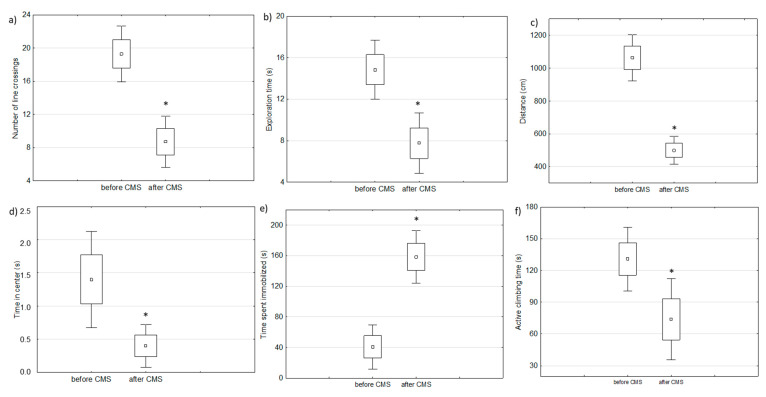
The results of behavioral changes after chronic mild stress protocol: (**a**–**d**) are parameters measured in an open filed test and (**e**,**f**) are parameters measured by a forced swim test (paired *t*-test, * defines *p* < 0.05).

Two-week chronic mild stress protocol resulted in depressive-like behavior measured by behavioral tests (FST and OFT) in analyzed rats as compared to the behavior of the same animals before starting the experiment (baseline) (Figure 2).

### 2.2. Brain Transcriptome Changes in the Model of Mania and Depression

**Figure 3:**
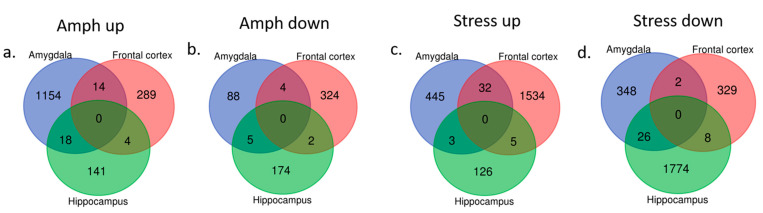
Differentially expressed genes (up- and downregulated) in three analyzed brain regions (amygdala, frontal cortex and hippocampus) in amphetamine-exposed (**a**,**b**, respectively) and stress-exposed rats (**c**,**d**, respectively) as compared to the control group.

Significant changes in the behavior following chronic mild stress protocol or amphetamine corresponded to specific gene expression profiles in three brain regions. In rats presenting manic-like behavior, we found 1283 genes differentially expressed in the amygdala, 637 genes in the frontal cortex and 344 genes in the hippocampus as compared to the control group (Figure 3a,b). The altered genes in the amygdala were mainly downregulated and significantly enriched in 23 Gene Ontology (GO) terms including olfactory receptor activity and chemical response to stimuli (Table 1). The genes differentially expressed in the frontal cortex and hippocampus were not significantly enriched in any GO terms or pathways.

In rats with depressive-like behavior, we found 856 differentially expressed genes (either up- or downregulated) in the amygdala, 1910 genes in the frontal cortex and 1942 genes in the hippocampus as compared to the control rats (Figure 3c,d).

The downregulated genes in the amygdala were enriched mainly in locomotor behavior and blood circulation (8 GO terms), whereas the upregulated genes were involved, among others, in animal organ morphogenesis, response to stimuli and signal transduction (8 GO terms) (Table 2). In the frontal cortex and hippocampus the differentially expressed genes (either upregulated or downregulated) were not significantly enriched in any GO terms or pathways.

### 2.3. Brain Transcriptome Changes during Chronic Lithium Administration

#### 2.3.1. Two-Week Lithium Administration

Lithium administration for two weeks resulted in significant changes in both, stress-exposed and manic-like rats in all analyzed brain regions. In rats presenting manic-like behavior and receiving lithium for two weeks, we found 4041 differentially expressed genes in the amygdala (all upregulated), 978 genes in the frontal cortex and 386 genes in the hippocampus. In the amygdala upregulated genes were enriched in 40 GO terms, mainly olfactory receptor activity, taste receptor and bitter taste receptor (Table 3). In the frontal cortex, most genes were significantly downregulated and involved in 11 GO terms, including olfactory receptor activity (Table 3). Genes differentially expressed in the hippocampus were not enriched in any GO terms.

Rats presenting depressive-like behavior showed differentially expressed 1650 genes in the amygdala, 481 genes in the frontal cortex and 304 genes in the hippocampus. The significantly altered genes in the amygdala were mainly downregulated and were involved in 206 GO terms, mainly associated with extracellular processes e.g., extracellular matrix organization (top 20 were listed in Table 4). In the frontal cortex differentially expressed genes were not significantly enriched in any GO terms between rats receiving lithium and control group. In the hippocampus, altered genes were significantly enriched in two GO terms, antigen processing and antigen binding (Table 4).

#### 2.3.2. Four-Week Lithium Administration

In rats presenting manic-like behavior and receiving lithium for 4 weeks, we observed 2831 genes differentially expressed in the amygdala, 1964 genes in the frontal cortex and 7412 genes in the hippocampus. In the amygdala, all genes were downregulated after 4 weeks on lithium and were enriched mainly in olfactory receptor activity and bitter taste (Table 5). In the frontal cortex, downregulated genes were enriched in 31 GO terms including, e.g., olfactory receptor activity (Table 5). In the hippocampus significantly upregulated genes were enriched in 57 GO terms (mainly response to chemical stimuli, bitter taste and olfactory receptor activity) (Table 5).

Four weeks of lithium treatment in stress-exposed rats, we observed that in the amygdala 778 genes were differentially expressed, in the frontal cortex 779 genes and in the hippocampus 307 genes. In the amygdala the genes were mainly upregulated and enriched in 17 GO terms, e.g., olfactory receptor activity (Table 6), in the frontal cortex altered genes were grouped in 24 GO terms (including nucleosome assembly, DNA-protein complex) and in the hippocampus differentially expressed genes were involved in extracellular matrix/space/region and collagen fibril organization (two upregulated GO terms) (Table 6).

#### 2.3.3. Molecular Pathways in the Brain during Chronic Lithium Treatment

**Figure 4:**
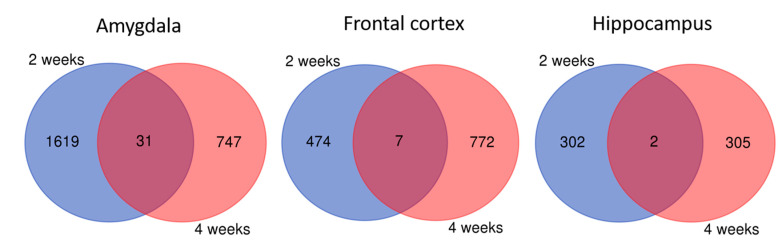
Genes differentially expressed in rats depressive-like after 2 weeks and 4 weeks on lithium treatment in the amygdala, frontal cortex and hippocampus. Shared genes between these time points were indicated by purple color.

When we combined together gene sets differentially expressed in rats with depressive-like behavior receiving lithium for two and four weeks, we observed only a few shared genes between these two time points either in the amygdala or frontal cortex and hippocampus as compared to the control rats (Figure 4). These genes were not enriched in any GO terms and pathways.

**Figure 5:**
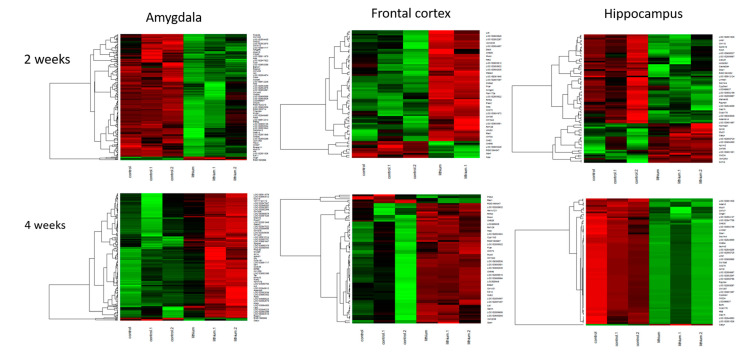
Gene-set enrichment analysis indicated that the genes from olfactory transduction pathway are influenced by chronic lithium administration in manic-like rats in analyzed brain areas (amygdala, frontal cortex and hippocampus).

In rats with manic-like behavior, we observed that chronic lithium treatment (2 and 4 weeks) significantly influenced the expression of the genes from the olfactory and taste transduction pathway (mainly different olfactory and taste receptors) and long non-coding RNAs in all three brain regions in rats receiving lithium as compared to water-receiving animals (Figure 5). Gene-set enrichment analysis showed that the shared genes between 2 and 4 weeks showed different gene expression profile: those upregulated after 2-week lithium treatment were downregulated after 4 weeks in the amygdala and frontal cortex. In the hippocampus, a 4-week lithium treatment upregulated all the genes.

### 2.4. Brain Transcriptome Changes in Lithium Responders

**Figure 6:**
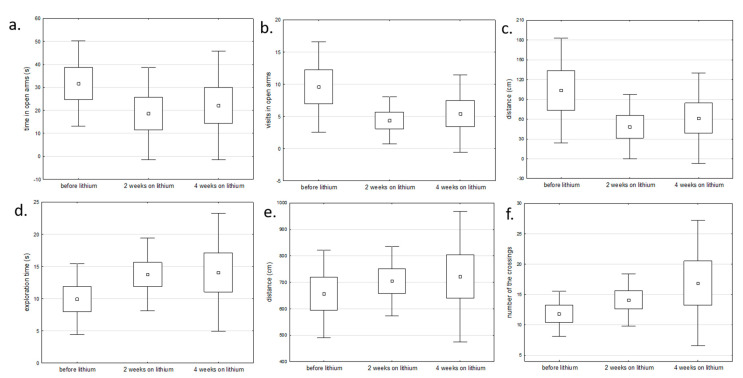
The comparison of behavioral changes before and after lithium administration in: (**a**–**c**) rats with manic-like behavior using elevated maze test and (**d**–**f**) rats with depressive-like behavior using open field test (one-way analysis of variance).

We also compared the gene expression profile before and after chronic lithium administration (2 and 4 weeks) in lithium responders. These rats showed changes in behavioral tests (open field test and elevated maze test) corresponding to reduced manic or depressive symptoms, e.g., reduced number of visits and time spent in open arms in manic rats after lithium and increased exploration time and number of line crossing in depressive rats after lithium (Figure 6).

Gene expression analysis in rats responding to lithium showed that in after chronic lithium administration gene expression was mainly upregulated in the amygdala and frontal cortex, but downregulated in the hippocampus in manic rats responding to lithium as compared to rats before lithium treatment. Interestingly, depressive rats responding to lithium showed the opposite trend, with mostly downregulated gene expression in the amygdala and frontal cortex, but upregulated in the hippocampus as compared to depressive rats before lithium. These genes were significantly enriched in GO terms related i.a. to olfactory receptor pathway, sensory perception of smell, G-protein coupled receptor and detection of stimulus (Table 7 and Table 8).

## 3. Discussion

The main observation of this study shows that the brain transcriptome is influenced by chronic lithium administration. The important finding is that the differentially expressed genes specific for the mania model are involved in olfactory receptor transduction and taste receptor pathways and show altered expression of long non-coding RNA genes.

Comparing depressive-like and manic-like animals, we found that in the depression model, the gene expression profile is more region-specific (different pathways regulated upon lithium in the amygdala, frontal cortex and hippocampus) and time-dependent (2 weeks versus 4 weeks). In the model of mania, on the other hand, lithium significantly affected olfactory receptor and bitter taste receptor pathways, independent of the brain region or time of lithium administration suggesting their role in antimanic action. The genes from these pathways also showed significantly altered expression in “manic” rats before lithium administration. Our observation is consistent with the previous human studies in bipolar disorder patients that presented several olfactory and gustatory dysfunctions [13] and the sensory enhancement and dysregulation of taste were often reported during an acute manic episode in BD patients [14].

Previous animal studies of lithium showed that mice chronically fed with lithium accumulated it mainly in neurogenic brain regions including olfactory bulb [15]. Similarly, chronic lithium effects in the neurogenic brain region (hippocampus) were also observed in human BD studies showing that lithium influenced hippocampal volume and structural plasticity in BD patients [16,17,18]. These findings suggest that chronic lithium treatment targets olfactory bulb and alters associated pathways (e.g., olfactory transduction).

On the contrary, in “depressive” rats we observed the upregulated expression of genes regulating olfactory receptor activity after 4-week lithium administration in the amygdala, which is consistent with the previous observations that bipolar patients during depressive episode experienced sensory blunting and olfactory deficits [14]. Thus chronic lithium treatment seems to restore olfactory function in rats presenting depressive-like behavior. Moreover, previous study showed that olfactory bulbectomy induced depressive-like behavior in rodents [19], in particular when taking into account the close anatomical links between the olfactory system and the brain circuits involved in memory [20] and emotion [21]. The recent studies reported the presence of sensory changes during mood swings in BD [22] and that olfactory assessment may be useful to screen unipolar and bipolar depression [23]. These genes involved in olfactory receptors activity were also differentially expressed in either manic and depressive rats responding to chronic lithium treatment and showed the opposite effect that also depended on tissue: in the amygdala they were upregulated after chronic lithium in mania, but downregulated in depression; in the hippocampus they showed downregulated expression in mania, but upregulated in depression after chronic lithium. This opposite gene expression profiles between amygdala and hippocampus after chronic lithium treatment may be further supported by results of the recent study that showed the opposite temporal profiles of protein expression between amygdala and hippocampal neurons in long-term response to acute stress [24].

Our findings from the animal model indicating the involvement of the taste receptor pathway were further supported by the observations in our clinical sample of bipolar and unipolar patients showing altered expression of taste receptor genes in peripheral blood leukocytes during depressive episode (Dmitrzak-Węglarz et al., under review). However, this pathway was not previously reported in the chronic lithium mechanism of action.

Another pathway significantly influenced by chronic lithium treatment in our animal mania model was bitter taste receptor pathway. Recent transcriptome study by Lee et al. [25] showed altered taste receptors gene expression during manic episodes. These genes included bitter taste receptors, TAS2R5 and TAS2R3, that were suggested as potential markers specific for the manic state [25]. These receptors were previously found downregulated (together with olfactory receptors) in the dorsolateral prefrontal cortex of schizophrenia postmortem brain tissues [26] and this downregulation influenced altered cognition. The taste alteration was also related to psychosocial and cognitive performance in bipolar patients [13]. However, the previous studies did not investigate the effects of mood stabilizers on taste-related gene expression. Our observation that chronic lithium influences the regulation of the olfactory and taste receptors’ pathway is supported by the previous case studies reporting long lasting impaired taste (dysgeusia) and smell (hyposmia) in lithium users: cluster headache patient [27] and bipolar patient [28].

Our study revealed that a number of differentially expressed genes influenced by chronic lithium treatment in the brain were long non-coding RNAs. The paper by ConLiGen showed significant associations for two long non-coding RNAs (lncRNAs) localized on chromosome 21 with lithium prophylactic efficacy supporting their involvement in modulating a clinical response [29]. Several studies reported that lncRNAs expression was dysregulated in psychiatric conditions, including BD [30] whereas Lee et al. found recently that lncRNAs were the predominant category showing upregulation during an acute manic episode [25]. Here, we report for the first time that lncRNAs expression is altered by chronic lithium treatment and suggest they may be regulators of antimanic lithium action.

Comparing the 2-week and 4-week gene expression profile, we found that the longer the treatment was, the more differentially expressed genes were observed, independent of the brain region. A previous study comparing microarrays from rat brains after 7 and 42 days of lithium treatment showed that, although plasma Li concentration reached therapeutic levels after 2 days of treatment, it required 2 weeks to reach therapeutic levels in the brain [11]. This finding suggested that the long-term lithium treatment and associated gene expression profile changes better represent the downstream Li effects, which are more relevant to its clinical effect. The other report in the rat fed for 21 days with lithium led to significant changes in the transcriptome in the frontal cortex [31].

To investigate the chronic lithium effect in the brain that mimics changes during the episode of depression or mania, we aimed to model mania and depression separately as the animal model of bipolar disorder does not exist [32]. Animal models are relevant platforms to evaluate the intracellular mechanisms in the brain associated with the pathogenesis of psychiatric disorders and drug studies, including lithium discovery [2,33,34]. In our study, we applied a chronic mild stress (CMS) protocol, the most extensively validated [35], to induce depressive-like behavior. Moreover, the CMS protocol was also suitable to model recurrent depression (a hallmark of bipolar disorder) [36]. The choice of amphetamine to mimic mania was supported by the previous preclinical studies that used repeated intraperitoneal injections of psychostimulants, such as amphetamine, to mimic acute manic episodes in rodents, including hyperactivity and risk-taking behavior due to increased sustained dopamine efflux [37,38,39,40,41,42]. Moreover, these behaviors were reversed by the administration of mood stabilizers, including lithium [40,43]. This chronic amphetamine-induced mania model showed recently good face, predictive and construct validity in modeling mania in Wistar rats [44,45,46,47].

## 4. Materials and Methods

### 4.1. Experimental Animals

**Figure 7:**
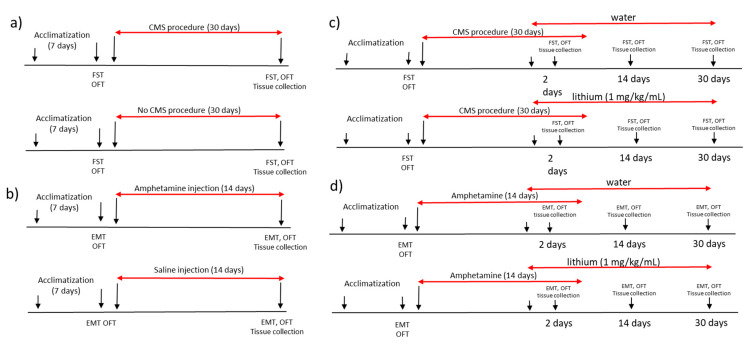
The experimental design of the study showing the (**a**) procedure of inducing manic-like behavior, (**b**) procedure of inducing depressive-like behavior, (**c**) lithium administration in rats with manic-like behavior and (**d**) lithium administration in animals showing depressive-like behavior. CMS—chronic mild stress protocol; EMT—elevated maze test, FST—forced swim test, OFT—open field test.

All experimental procedures were performed in agreement with 3R rule and the study was approved by local ethical committee Poznan University of Life Sciences, Poland (agreement no. 22/2017, 23 June 2017). We used male Wistar rats with the baseline weight of 180 ± 10 g. The rats were housed five animals per cage with food and water available ad libitum and were maintained in a 12 h light/dark cycle (lights on at 7:00 a.m.) at a temperature of 22 ± 1 °C. All experiments were performed at the same time each day to avoid circadian variations. The animals were kept for one week of acclimatization and then were randomly divided into experimental groups with 5 animals in each group (amphetamine-exposed and control group, chronic mild stress-exposed and non-stressed rats; amphetamine-exposed rats receiving lithium or water and stress-exposed rats receiving lithium or water). After the acclimatization period, animals underwent baseline behavioral tests. The experimental design was shown in Figure 7.

### 4.2. Animal Model of Mania

Manic-like behavior was induced by daily intraperitoneal (i.p) injections of dextroamphetamine (d-AMPH) 2 mg/kg for 2 weeks as described previously [37,42]. The control group were animals receiving daily intraperitoneal injection with saline (0.9% NaCl, 1 mL/kg) for 14 days. After seven days, the behavior of all animals was assessed (Figure 7).

### 4.3. Animal Model of Depression

Animal model of depression was developed by the chronic mild stress protocol (CMS) as described previously [48]. To induce depressive behavior the animals were exposed for 4 weeks to different stress stimuli according to the CMS protocol described by Papp [49]. The control group were rats kept in standard conditions for 4 weeks that were not exposed to CMS. After two weeks of the start of the experiment, behavior of all animals was assessed (Figure 7).

### 4.4. Lithium Administration

The animals for the lithium study, after one week of amphetamine injections or two weeks of the chronic mild stress protocol and behavioral assessment, were randomly divided into lithium treated amphetamine-exposed group (*n* = 15) and amphetamine-exposed control group (rats receiving water, *n* = 15) and lithium-treated stress-exposed group (*n* = 15) and a stress-exposed control group (rats receiving water, *n* = 15). The animals were receiving the daily lithium solution in the dose of 1 mg/kg body mass or the same amount of water into the mouth with a syringe [50]. To minimize the side effects of lithium on kidney function, a saline bottle was provided in all lithium and control cages, as previously reported [51]. To assess the short term and long term lithium effects on brain transcriptome, the animals (*n* = 15 at each time in each group) were sacrificed by decapitation without euthanasia at two time points of chronic lithium administration, 14 and 30 days (long-term effect) after the first dose. The each control group (for stress-exposed and amphetamine-exposed rats) consisted of animals receiving water (Figure 7).

### 4.5. Behaviour Assessment

All the behavioral tests were performed during the light phase (between 08:00 a.m. and 12:00 p.m.), at room temperature in a separate quiet room. A blind evaluator assessed all behavioral parameters. To assess depressive-like or manic-like behavior we used a forced-swim test, open field test and elevated maze test at baseline and after induction of stress-induced depression or amphetamine-induced mania.

A forced swim test was performed to measure despair behavior and the hopelessness in the animal [52,53]. Each rat was placed in a glass cylinder 40 cm tall filled with water to a depth of 30 cm (24 ± 1 °C) so the rats could not support themselves by touching the bottom. Two swimming sessions were conducted: a 15-min pretest followed by a 5-min test 24 h later. For each animal, we recorded time spent immobilized (no additional activity other than that required to keep the rat’s head above the water) and active climbing time (upward-directed movements of the forepaws along the side of the swim chamber. The data were analyzed using the video tracking system (Videomot2, AnimaLab, Poznan, Poland). The water in the cylinders was replaced after each trial to remove urine or feces and to avoid confounding results.

Open field test was performed to evaluate the spontaneous locomotory activity in a new environment and exploration [54,55,56]. The rat was placed in the middle of the plastic box measuring 100 cm in diameter with 50-cm walls, divided into 25 squares of 20 × 20-cm size on the bottom (AnimaLab, Poland) and allowed to explore it for 5 min. We recorded the following parameters: time spent immobilized, time spent in center, total distance and exploration time and analyzed the data with the video tracking system (Videomot2, AnimaLab, Poland). After the end of each test, the open field was cleaned to remove any remaining materials (such as feces, urine or smell) that could interfere with the test results.

The elevated maze test was used to test the anxiety level and risk-taking behavior in the rats [42]. The apparatus consisted of two opposite open arms, two opposite closed arms and a central platform connecting the four arms. The maze was propped up 50 cm away from the ground by the maze feet. The test room was maintained with constant temperature, humidity and illumination. The rats were placed in the central platform facing one of the open arms. The number of entries into the open and closed arms and the total time spent in each arm during a 5-min exploration period were recorded by the video tracking system (Videomot2, AnimaLab, Poznań, Poland). After the end of each test, the open field was cleaned to remove any remaining materials (such as feces, urine or smell) that could interfere with the test results.

### 4.6. Microarray-Based Gene Expression Analysis

The different regions of brain tissue (amygdala, hippocampus, prefrontal cortex and hypothalamus) were collected immediately after decapitation (without anesthesia). The skull was opened, and the cerebral content was excised and rapidly dissected on a chilled Petri dish. The frontal cortex, hippocampus and amygdala were isolated and cleaned from the subcortical structures and white matter, immediately snap frozen in liquid nitrogen and kept frozen in −80 °C for further processing. Frozen tissues were used for RNA extraction using Nucleospin RNA/Protein kit (Macherey Nagel, Dylan, Germany). RNA integrity (RIN) was assessed using Tape Station 2200 (Agilent) and RNA concentration was measured using fluorimeter (Quantus, Promega). Total RNA from amygdala, hippocampus and frontal cortex in the starting amount of 50 ng was used for microarray experiments. We used SurePrint G3 Rat Gene Expression v2 Microarray Kit in format 8 × 60 k and one-color Low Input Quick Amp Labeling Kit (Agilent Technologies, Cedar Creek, TX, USA) to analyze gene expression profile following the standard protocol provided by the manufacturer. The hybridization signals were detected with SureScan Dx Microarray Scanner (Agilent Technologies, Santa Clara, CA, USA). The images obtained after scanning were analyzed with Agilent Feature extraction software v.12.0.3.1.

### 4.7. Statistical Analysis

Behavioral data were analyzed using a *t*-test for paired samples after checking normality of data distribution using the Shapiro–Wilk test. A cut-off value *p* < 0.05 was regarded as significant. Data were shown as mean ± SEM in figures and text if not otherwise stated. For behavioral measurements and gene expression data, 5 animals per experimental group were sufficient to achieve power of 80% (as calculated by the G-power calculator available at https://stats.idre.ucla.edu/other/gpower). To conform to the 3R’s rule, we included the minimal required number of animals (*n* = 5) in the study.

Bioinformatic analysis of gene expression data was analyzed with the use of Gene Spring 14.9 software (Agilent Technologies, Santa Clara, CA, USA). To identify differentially expressed genes, moderated t-statistics from the empirical Bayes method on normalized fluorescence signal was used for the fold of change (FC) calculation. FC was calculated in relation to the adequate control group. The list of significantly differentially expressed transcripts (*p* < 0.05 and FC > 2.0) was generated using statistical filtering (moderated *t*-test with multiple test correction FDR). Then, separate lists of up- and downregulated genes were used for functional analysis to identify Gene Ontology (GO) terms and pathways significantly enriched by the set of differentially expressed genes, either upregulated or downregulated (*p* < 0.05). Gene Ontology and pathway analyses were done in Gene Spring.

To identify the genes and pathways shared during lithium administration, we used Venn diagrams using a tool freely available at: http://bioinformatics.psb.ugent.be/webtools/Venn. For shared genes, we used G:Profiler tool (available at: https://biit.cs.ut.ee/gprofiler/gost) to perform functional enrichment analysis (gene set enrichment analysis) including GO and pathways from KEGG Reactome and Wiki Pathways [57]. Heat maps were drawn using the Heatmapper tool available at: http://www.heatmapper.ca.

## 5. Conclusions

We reported here for the first time that genes regulating olfactory and taste receptor pathways and long non-coding RNAs, that were implicated in BD pathogenesis, might be targeted by chronic lithium treatment in animals presenting manic-like behavior. Further functional studies of these pathways are warranted to elucidate the exact therapeutic molecular mechanism of lithium.

## Figures and Tables

**Table 1:** Gene Ontology (GO) terms enriched from significantly downregulated genes between rats with manic-like behavior and control rats in the amygdala.

GO Accession	GO Term	Corr *p*	No. Genes
GO:0007186	G-protein coupled receptor signaling pathway	0.000	117
GO:0007600	sensory perception	0.000	103
GO:0051606	detection of stimulus	0.000	88
GO:0050906	detection of stimulus involved in sensory perception	0.000	84
GO:0038023	signaling receptor activity	0.000	120
GO:0060089	molecular transducer activity	0.000	120
GO:0009593	detection of chemical stimulus	0.000	81
GO:0050907	detection of chemical stimulus involved in sensory perception	0.000	79
GO:0004888	transmembrane signaling receptor activity	0.000	111
GO:0004930	G-protein coupled receptor activity	0.000	93
GO:0004871	signal transducer activity	0.000	126
GO:0007606	sensory perception of chemical stimulus	0.000	82
GO:0004984	olfactory receptor activity	0.000	74
GO:0050911	detection of chemical stimulus involved in sensory perception of smell	0.000	74
GO:0007608	sensory perception of smell	0.000	75
GO:0007165	signal transduction	0.000	201
GO:0003008	system process	0.000	123
GO:0050877	nervous system process	0.000	106
GO:0023052	signaling	0.000	209
GO:0007154	cell communication	0.000	212
GO:0050896	response to stimulus	0.001	287
GO:0051716	cellular response to stimulus	0.004	238
GO:0005833	hemoglobin complex	0.019	5

**Table 2:** GO terms significantly enriched from differentially expressed genes between rats with depressive-like behavior and control group in the amygdala.

GO Accession	GO Term	Corr *p*	No. Genes
	**Downregulated**		
GO:0007610	behavior	0.002	26
GO:0007188	adenylate cyclase-modulating G-protein coupled receptor signaling pathway	0.002	12
GO:0007187	G-protein coupled receptor signaling pathway, coupled to cyclic nucleotide second messenger	0.003	12
GO:0044459	plasma membrane part	0.004	53
GO:0003013	circulatory system process	0.004	17
GO:0008015	blood circulation	0.004	17
GO:0007626	locomotory behavior	0.004	14
GO:0007193	adenylate cyclase-inhibiting G-protein coupled receptor signaling pathway	0.037	7
	**Upregulated**		
GO:0023052	signaling	0.000	102
GO:0007154	cell communication	0.000	102
GO:0007165	signal transduction	0.000	95
GO:0038023	signaling receptor activity	0.001	55
GO:0060089	molecular transducer activity	0.001	55
GO:0004888	transmembrane signaling receptor activity	0.001	52
GO:0004871	signal transducer activity	0.006	57
GO:0007186	G-protein coupled receptor signaling pathway	0.006	49
GO:0003008	system process	0.006	57
GO:0051606	detection of stimulus	0.017	37
GO:0050896	response to stimulus	0.022	126
GO:0004930	G-protein coupled receptor activity	0.028	40
GO:0009593	detection of chemical stimulus	0.028	34
GO:0050906	detection of stimulus involved in sensory perception	0.045	34
GO:0009653	anatomical structure morphogenesis	0.050	45

**Table 3:** Differentially expressed genes enriched in GO terms in amphetamine-exposed rats after two weeks of lithium treatment.

GO Accession	GO Term	Corr *p*	No. Genes
	**Amygdala upregulated**		
GO:0038023	signaling receptor activity	0.000	367
GO:0060089	molecular transducer activity	0.000	367
GO:0004888	transmembrane signaling receptor activity	0.000	352
GO:0007186	G-protein coupled receptor signaling pathway	0.000	344
GO:0007600	sensory perception	0.000	310
GO:0004930	G-protein coupled receptor activity	0.000	299
GO:0007606	sensory perception of chemical stimulus	0.000	285
GO:0051606	detection of stimulus	0.000	281
GO:0050906	detection of stimulus involved in sensory perception	0.000	273
GO:0009593	detection of chemical stimulus	0.000	268
GO:0050907	detection of chemical stimulus involved in sensory perception	0.000	264
GO:0007608	sensory perception of smell	0.000	256
GO:0004984	olfactory receptor activity	0.000	247
GO:0050911	detection of chemical stimulus involved in sensory perception of smell	0.000	247
GO:0004871	signal transducer activity	0.000	379
GO:0050877	nervous system process	0.000	330
GO:0003008	system process	0.000	363
GO:0031224	intrinsic component of membrane	0.000	640
GO:0016021	integral component of membrane	0.000	627
GO:0007165	signal transduction	0.000	509
GO:0042221	response to chemical	0.000	507
GO:0044425	membrane part	0.000	687
GO:0005886	plasma membrane	0.000	521
GO:0005549	odorant binding	0.000	68
GO:0071944	cell periphery	0.000	528
GO:0023052	signaling	0.000	523
GO:0007154	cell communication	0.000	533
GO:0032501	multicellular organismal process	0.000	669
GO:0050896	response to stimulus	0.000	731
GO:0004252	serine-type endopeptidase activity	0.000	44
GO:0051716	cellular response to stimulus	0.000	597
GO:0008236	serine-type peptidase activity	0.000	44
GO:0017171	serine hydrolase activity	0.000	44
GO:0008527	taste receptor activity	0.000	15
GO:0050912	detection of chemical stimulus involved in sensory perception of taste	0.001	17
GO:0050913	sensory perception of bitter taste	0.002	16
GO:0033038	bitter taste receptor activity	0.002	13
GO:0001580	detection of chemical stimulus involved in sensory perception of bitter taste	0.004	15
GO:0004175	endopeptidase activity	0.005	61
GO:0050909	sensory perception of taste	0.007	19
	**Frontal cortex downregulated**		
GO:0038023	signaling receptor activity	0.022	80
GO:0060089	molecular transducer activity	0.022	80
GO:0007608	sensory perception of smell	0.022	52
GO:0004984	olfactory receptor activity	0.022	50
GO:0050911	detection of chemical stimulus involved in sensory perception of smell	0.022	50
GO:0009593	detection of chemical stimulus	0.026	52
GO:0050907	detection of chemical stimulus involved in sensory perception	0.026	51
GO:0051606	detection of stimulus	0.029	55
GO:0007606	sensory perception of chemical stimulus	0.033	54
GO:0050906	detection of stimulus involved in sensory perception	0.033	52
GO:0004888	transmembrane signaling receptor activity	0.036	72

**Table 4:** Differentially expressed genes enriched in GO terms in stress-exposed rats after two weeks of lithium treatment.

GO Accession	GO Term	Corr *p*	No. Genes
	**Amygdala (top 20 out of 206)**		
GO:0005578	proteinaceous extracellular matrix	0.000	41
GO:0031012	extracellular matrix	0.000	34
GO:0005576	extracellular region	0.000	117
GO:0044421	extracellular region part	0.000	108
GO:0005615	extracellular space	0.000	104
GO:0044420	extracellular matrix component	0.000	19
GO:0009888	tissue development	0.000	62
GO:0030198	extracellular matrix organization	0.000	21
GO:0009653	anatomical structure morphogenesis	0.000	68
GO:0043062	extracellular structure organization	0.000	21
GO:0007275	multicellular organism development	0.000	112
GO:0030199	collagen fibril organization	0.000	11
GO:0072001	renal system development	0.000	24
GO:0005581	collagen trimer	0.000	13
GO:0032502	developmental process	0.000	122
GO:0048513	animal organ development	0.000	87
GO:0048646	anatomical structure formation involved in morphogenesis	0.000	39
GO:0009887	animal organ morphogenesis	0.000	41
GO:0048731	system development	0.000	104
	**Hippocampus**		
GO:0002474	antigen processing and presentation of peptide antigen via MHC class I	0.024	4
GO:0042605	peptide antigen binding	0.024	4

**Table 5:** Differentially expressed genes enriched in GO terms in amphetamine-exposed rats after four weeks of lithium treatment.

GO Accession	GO Term	Corr *p*	No. Genes
**Amygdala downregulated**			
GO:0038023	signaling receptor activity	0.000	228
GO:0060089	molecular transducer activity	0.000	228
GO:0004888	transmembrane signaling receptor activity	0.000	212
GO:0007608	sensory perception of smell	0.000	149
GO:0004984	olfactory receptor activity	0.000	144
GO:0050911	detection of chemical stimulus involved in sensory perception of smell	0.000	144
GO:0007606	sensory perception of chemical stimulus	0.000	159
GO:0007600	sensory perception	0.000	186
GO:0050907	detection of chemical stimulus involved in sensory perception	0.000	147
GO:0050906	detection of stimulus involved in sensory perception	0.000	151
GO:0051606	detection of stimulus	0.000	157
GO:0009593	detection of chemical stimulus	0.000	147
GO:0004930	G-protein coupled receptor activity	0.000	172
GO:0004871	signal transducer activity	0.000	237
GO:0007186	G-protein coupled receptor signaling pathway	0.000	196
GO:0050877	nervous system process	0.000	199
GO:0031224	intrinsic component of membrane	0.000	451
GO:0016021	integral component of membrane	0.000	441
GO:0003008	system process	0.000	220
GO:0044425	membrane part	0.002	488
GO:0005886	plasma membrane	0.003	365
GO:0023052	signaling	0.007	366
GO:0071944	cell periphery	0.008	369
GO:0007165|GO:0023033	signal transduction	0.009	346
GO:0007154	cell communication	0.011	373
GO:0005549	odorant binding	0.020	40
**Frontal cortex downregulated**			
GO:0007606	sensory perception of chemical stimulus	0.000	154
GO:0004984	olfactory receptor activity	0.000	140
GO:0050911	detection of chemical stimulus involved in sensory perception of smell	0.000	140
GO:0009593	detection of chemical stimulus	0.000	145
GO:0050907	detection of chemical stimulus involved in sensory perception	0.000	143
GO:0007608	sensory perception of smell	0.000	142
GO:0007600	sensory perception	0.000	171
GO:0050906	detection of stimulus involved in sensory perception	0.000	144
GO:0051606	detection of stimulus	0.000	148
GO:0004888	transmembrane signaling receptor activity	0.000	183
GO:0004930	G-protein coupled receptor activity	0.000	154
GO:0038023	signaling receptor activity	0.000	190
GO:0060089	molecular transducer activity	0.000	190
GO:0050877	nervous system process	0.000	177
GO:0007186	G-protein coupled receptor signaling pathway	0.000	172
GO:0004871	signal transducer activity	0.000	197
GO:0003008	system process	0.000	195
GO:0031224	intrinsic component of membrane	0.000	340
GO:0016021	integral component of membrane	0.000	334
GO:0071944	cell periphery	0.000	287
GO:0005886	plasma membrane	0.000	281
GO:0042221	response to chemical	0.000	269
GO:0044425	membrane part	0.000	364
GO:0032501	multicellular organismal process	0.001	352
GO:0005549	odorant binding	0.001	34
GO:0036156	inner dynein arm	0.006	4
GO:0007165	signal transduction	0.006	252
GO:0004252	serine-type endopeptidase activity	0.019	22
GO:0023052	signaling	0.030	261
GO:0007154	cell communication	0.030	267
GO:0008236	serine-type peptidase activity	0.043	23
**Hippocampus downregulated**			
GO:0001580	detection of chemical stimulus involved in sensory perception of bitter taste	0.000	1394
GO:0001594	trace-amine receptor activity	0.000	1323
GO:0003008	system process	0.000	1316
GO:0004252	serine-type endopeptidase activity	0.000	1313
GO:0004866	endopeptidase inhibitor activity	0.000	1212
GO:0004867	serine-type endopeptidase inhibitor activity	0.000	1119
GO:0004869	cysteine-type endopeptidase inhibitor activity	0.000	1105
GO:0004871	signal transducer activity	0.000	1083
GO:0004888	transmembrane signaling receptor activity	0.000	1062
GO:0004930	G-protein coupled receptor activity	0.000	1053
GO:0004984	olfactory receptor activity	0.000	1037
GO:0005179	hormone activity	0.000	888
GO:0005549	odorant binding	0.000	877
GO:0005886	plasma membrane	0.000	877
GO:0007154	cell communication	0.000	850
GO:0007165	signal transduction	0.000	845
GO:0007186	G-protein coupled receptor signaling pathway	0.000	835
GO:0007600	sensory perception	0.000	791
GO:0007606	sensory perception of chemical stimulus	0.000	769
GO:0007608	sensory perception of smell	0.000	740
GO:0008527	taste receptor activity	0.000	720
GO:0009593	detection of chemical stimulus	0.000	697
GO:0010466	negative regulation of peptidase activity	0.000	684
GO:0010951	negative regulation of endopeptidase activity	0.000	675
GO:0016020	membrane	0.000	673
GO:0016021	integral component of membrane	0.000	652
GO:0016503	pheromone receptor activity	0.000	644
GO:0017171	serine hydrolase activity	0.000	644
GO:0019236	response to pheromone	0.000	173
GO:0023052	signaling	0.000	1413
GO:0030414	peptidase inhibitor activity	0.000	1507
GO:0030545	receptor regulator activity	0.000	41
GO:0031224	intrinsic component of membrane	0.000	41
GO:0032501	multicellular organismal process	0.000	1583
GO:0033038	bitter taste receptor activity	0.000	27
GO:0038023	signaling receptor activity	0.000	24
GO:0042221	response to chemical	0.000	1644
GO:0042742	defense response to bacterium	0.000	29
GO:0044425	membrane part	0.000	1726
GO:0048018	receptor ligand activity	0.000	26
GO:0050789	regulation of biological process	0.000	26
GO:0050794	regulation of cellular process	0.000	32
GO:0050877	nervous system process	0.000	57
GO:0050896	response to stimulus	0.000	54
GO:0050906	detection of stimulus involved in sensory perception	0.000	54
GO:0050907	detection of chemical stimulus involved in sensory perception	0.000	13
GO:0050909	sensory perception of taste	0.000	40
GO:0050911	detection of chemical stimulus involved in sensory perception of smell	0.001	90
GO:0050912	detection of chemical stimulus involved in sensory perception of taste	0.001	57
GO:0050913	sensory perception of bitter taste	0.001	94
GO:0051606	detection of stimulus	0.001	63
GO:0051716	cellular response to stimulus	0.002	60
GO:0060089	molecular transducer activity	0.010	23
GO:0061134	peptidase regulator activity	0.011	54
GO:0061135	endopeptidase regulator activity	0.014	49
GO:0065007	biological regulation	0.025	28
GO:0071944	cell periphery	0.043	53

**Table 6:** Differentially expressed genes enriched in GO terms in stress-exposed rats after four weeks of lithium treatment.

GO Accession	GO Term	Corr *p*	No. Genes
	**Amygdala upregulated**		
GO:0038023	signaling receptor activity	0.000	63
GO:0060089	molecular transducer activity	0.000	63
GO:0004888	transmembrane signaling receptor activity	0.000	59
GO:0007600	sensory perception	0.000	54
GO:0050907	detection of chemical stimulus involved in sensory perception	0.000	43
GO:0007606	sensory perception of chemical stimulus	0.000	45
GO:0009593	detection of chemical stimulus	0.000	43
GO:0051606	detection of stimulus	0.000	45
GO:0050906	detection of stimulus involved in sensory perception	0.000	43
GO:0007608	sensory perception of smell	0.000	41
GO:0004984	olfactory receptor activity	0.000	40
GO:0050911	detection of chemical stimulus involved in sensory perception of smell	0.000	40
GO:0007186	G-protein coupled receptor signaling pathway	0.000	56
GO:0004871	signal transducer activity	0.002	63
GO:0003008	system process	0.003	63
GO:0050877	nervous system process	0.003	55
GO:0004930	G-protein coupled receptor activity	0.003	46
	**Frontal cortex upregulated**		
GO:0000786	nucleosome	0.000	5
GO:0006334	nucleosome assembly	0.000	5
GO:0044815	DNA packaging complex	0.000	5
GO:0045653	negative regulation of megakaryocyte differentiation	0.000	3
GO:0031497	chromatin assembly	0.000	5
GO:0006333	chromatin assembly or disassembly	0.000	5
GO:0034728	nucleosome organization	0.000	5
GO:0006323	DNA packaging	0.000	5
GO:0065004	protein-DNA complex assembly	0.000	5
GO:0006335	DNA replication-dependent nucleosome assembly	0.000	3
GO:0034723	DNA replication-dependent nucleosome organization	0.000	3
GO:0032993	protein-DNA complex	0.000	5
GO:0071824	protein-DNA complex subunit organization	0.000	5
GO:0000788	nuclear nucleosome	0.000	3
GO:0006336	DNA replication-independent nucleosome assembly	0.000	3
GO:0045652	regulation of megakaryocyte differentiation	0.000	3
GO:0034724	DNA replication-independent nucleosome organization	0.000	3
GO:0071103	DNA conformation change	0.001	5
GO:0051290	protein heterotetramerization	0.002	3
GO:0051291	protein heterooligomerization	0.006	4
GO:0030492	hemoglobin binding	0.008	2
GO:0006352	DNA-templated transcription. initiation	0.018	3
GO:0045638	negative regulation of myeloid cell differentiation	0.018	3
GO:0000785	chromatin	0.047	5
**Hippocampus upregulated**			
GO:0005578	proteinaceous extracellular matrix	0.022	10
GO:0031012	extracellular matrix	0.009	13

**Table 7:** Differentially expressed genes enriched in GO terms in amphetamine-exposed rats responding to chronic lithium in comparison to rats before lithium.

Go Accession	GO Term	Corr *p*	No. Genes
**upregulated in the amygdala**			
GO:0007606	sensory perception of chemical stimulus	0.000	232
GO:0009593	detection of chemical stimulus	0.000	220
GO:0050907	detection of chemical stimulus involved in sensory perception	0.000	216
GO:0051606	detection of stimulus	0.000	232
GO:0050906	detection of stimulus involved in sensory perception	0.000	220
GO:0004930	G-protein coupled receptor activity	0.000	252
GO:0007608	sensory perception of smell	0.000	210
GO:0004984	olfactory receptor activity	0.000	205
GO:0050911	detection of chemical stimulus involved in sensory perception of smell	0.000	205
GO:0007186	G-protein coupled receptor signaling pathway	0.000	291
GO:0007600	sensory perception	0.000	261
GO:0004888	transmembrane signaling receptor activity	0.000	293
GO:0038023	signaling receptor activity	0.000	306
GO:0060089	molecular transducer activity	0.000	306
GO:0004871	signal transducer activity	0.000	317
GO:0050877	nervous system process	0.000	275
GO:0003008	system process	0.000	306
GO:0031224	intrinsic component of membrane	0.000	556
GO:0016021	integral component of membrane	0.000	548
GO:0007165	signal transduction	0.000	436
GO:0032501	multicellular organismal process	0.000	596
GO:0007154	cell communication	0.000	464
GO:0023052	signaling	0.000	452
GO:0044425	membrane part	0.000	596
GO:0005886	plasma membrane	0.000	447
GO:0042221	response to chemical	0.000	429
GO:0071944	cell periphery	0.000	455
GO:0030414	peptidase inhibitor activity	0.000	35
GO:0005549	odorant binding	0.001	50
GO:0004252	serine-type endopeptidase activity	0.001	34
GO:0004866	endopeptidase inhibitor activity	0.001	32
GO:0061135	endopeptidase regulator activity	0.003	32
GO:0061134	peptidase regulator activity	0.005	35
GO:0010466	negative regulation of peptidase activity	0.011	38
GO:0008236	serine-type peptidase activity	0.019	34
GO:0017171	serine hydrolase activity	0.025	34
GO:0050896	response to stimulus	0.026	631
GO:003024	carbohydrate binding	0.041	38
GO:0010951	negative regulation of endopeptidase activity	0.044	35
GO:0004869	cysteine-type endopeptidase inhibitor activity	0.046	15
**upregulated in the frontal cortex**			
GO:0004930	G-protein coupled receptor activity	0.000	44
GO:0004888	transmembrane signaling receptor activity	0.000	49
GO:0007186	G-protein coupled receptor signaling pathway	0.000	49
GO:0038023	signaling receptor activity	0.001	51
GO:0060089	molecular transducer activity	0.001	51
GO:0004871	signal transducer activity	0.003	53
**downregulated in the hippocampus**			
GO:0007186	G-protein coupled receptor signaling pathway	0.000	87
GO:0004888	transmembrane signaling receptor activity	0.000	85
GO:0038023	signaling receptor activity	0.000	89
GO:0060089	molecular transducer activity	0.000	89
GO:0004930	G-protein coupled receptor activity	0.000	68
GO:0004871|	signal transducer activity	0.000	92
GO:0007600	sensory perception	0.003	67
GO:0050906	detection of stimulus involved in sensory perception	0.007	54
GO:0050907	detection of chemical stimulus involved in sensory perception	0.008	52
GO:0051606	detection of stimulus	0.009	56
GO:0007606	sensory perception of chemical stimulus	0.011	55
GO:0009593	detection of chemical stimulus	0.011	52
GO:0003008	system process	0.011	86
GO:0004984	olfactory receptor activity	0.014	49
GO:0050911	detection of chemical stimulus involved in sensory perception of smell	0.014	49
GO:0007608	sensory perception of smell	0.031	49
GO:0050877	nervous system process	0.071	71

**Table 8:** Differentially expressed genes enriched in GO terms in stress-exposed rats responding to chronic lithium treatment in comparison to rats before lithium.

Go Accession	GO Term	Corr *p*	No. Genes
**downregulated in the amygdala**			
GO:0044425	membrane part	0.000	1150
GO:0032501|	multicellular organismal process	0.000	1107
GO:0031224	intrinsic component of membrane	0.000	1098
GO:0016021	integral component of membrane	0.000	1090
GO:0007154	cell communication	0.000	941
GO:0023052	signaling	0.000	930
GO:0007165	signal transduction	0.000	909
GO:0042221	response to chemical	0.000	895
GO:0071944	cell periphery	0.000	881
GO:0005886	plasma membrane	0.000	874
GO:0004871	signal transducer activity	0.000	773
GO:0038023	signaling receptor activity	0.000	762
GO:0060089	molecular transducer activity	0.000	762
GO:0004888	transmembrane signaling receptor activity	0.000	749
GO:0007186	G-protein coupled receptor signaling pathway	0.000	736
GO:0003008	system process	0.000	720
GO:0050877	nervous system process	0.000	683
GO:0007600	sensory perception	0.000	667
GO:0004930	G-protein coupled receptor activity	0.000	652
GO:0007606	sensory perception of chemical stimulus	0.000	632
GO:0051606	detection of stimulus	0.000	593
GO:0050906	detection of stimulus involved in sensory perception	0.000	586
GO:0009593	detection of chemical stimulus	0.000	578
GO:0050907	detection of chemical stimulus involved in sensory perception	0.000	578
GO:0007608	sensory perception of smell	0.000	565
GO:0004984	olfactory receptor activity	0.000	556
GO:0050911	detection of chemical stimulus involved in sensory perception of smell	0.000	556
GO:0005549	odorant binding	0.000	164
GO:0051716	cellular response to stimulus	0.000	1009
GO:0050896	response to stimulus	0.000	1174
GO:0016503	pheromone receptor activity	0.000	48
GO:0019236	response to pheromone	0.000	47
GO:0016020	membrane	0.000	1232
GO:0050789	regulation of biological process	0.000	1346
GO:0065007	biological regulation	0.000	1417
GO:0050794	regulation of cellular process	0.000	1286
GO:0033038	bitter taste receptor activity	0.000	19
GO:0008527	taste receptor activity	0.000	20
GO:0001580	detection of chemical stimulus involved in sensory perception of bitter taste	0.000	21
GO:0050909	sensory perception of taste	0.000	27
GO:0050912	detection of chemical stimulus involved in sensory perception of taste	0.000	22
GO:0050913	sensory perception of bitter taste	0.000	21
GO:0030414	peptidase inhibitor activity	0.000	47
GO:0004866	endopeptidase inhibitor activity	0.000	44
GO:0061135	endopeptidase regulator activity	0.001	44
GO:0004252	serine-type endopeptidase activity	0.001	45
GO:0008227	G-protein coupled amine receptor activity	0.003	22
GO:0017171	serine hydrolase activity	0.003	49
GO:0008236	serine-type peptidase activity	0.004	48
GO:0061134	peptidase regulator activity	0.006	47
GO:0004867	serine-type endopeptidase inhibitor activity	0.016	25
GO:0019373	epoxygenase P450 pathway	0.030	10
GO:0010466	negative regulation of peptidase activity	0.037	50
**downregulated in the frontal cortex**			
GO:0004930	G-protein coupled receptor activity	0.000	50
GO:0007606	sensory perception of chemical stimulus	0.000	45
GO:0009593	detection of chemical stimulus	0.000	43
GO:0038023	signaling receptor activity	0.000	61
GO:0060089	molecular transducer activity	0.000	61
GO:0007600	sensory perception	0.000	51
GO:0050907	detection of chemical stimulus involved in sensory perception	0.000	42
GO:0007608	sensory perception of smell	0.000	41
GO:0004984	olfactory receptor activity	0.000	40
GO:0050911	detection of chemical stimulus involved in sensory perception of smell	0.000	40
GO:0051606	detection of stimulus	0.000	44
GO:0050906	detection of stimulus involved in sensory perception	0.000	42
GO:0004888	transmembrane signaling receptor activity	0.000	56
GO:0007186	G-protein coupled receptor signaling pathway	0.001	54
GO:0003008	system process	0.001	63
GO:0004871	signal transducer activity	0.002	62
GO:0050877	nervous system process	0.002	54
GO:0016021	integral component of membrane	0.006	112
GO:0031224	intrinsic component of membrane	0.007	113
**upregulated in the hippocampus**			
GO:0004930	G-protein coupled receptor activity	0.000	118
GO:0007606	sensory perception of chemical stimulus	0.000	108
GO:0050907	detection of chemical stimulus involved in sensory perception	0.000	102
GO:0009593	detection of chemical stimulus	0.000	103
GO:0050906	detection of stimulus involved in sensory perception	0.000	104
GO:0004984	olfactory receptor activity	0.000	98
GO:0050911	detection of chemical stimulus involved in sensory perception of smell	0.000	98
GO:0051606	detection of stimulus	0.000	105
GO:0007608	sensory perception of smell	0.000	98
GO:0004888	transmembrane signaling receptor activity	0.000	129
GO:0038023	signaling receptor activity	0.000	135
GO:0060089	molecular transducer activity	0.000	135
GO:0007186	G-protein coupled receptor signaling pathway	0.000	127
GO:0007600	sensory perception	0.000	115
GO:0004871	signal transducer activity	0.000	136
GO:0050877	nervous system process	0.000	119
GO:0003008	system process	0.000	127
GO:0016021	integral component of membrane	0.000	217
GO:0031224	intrinsic component of membrane	0.000	219
GO:0007165	signal transduction	0.000	173
GO:0023052	signaling	0.000	178
GO:0044425	membrane part	0.000	225
GO:0007154	cell communication	0.000	179
GO:0042221	response to chemical	0.000	167
GO:0071944	cell periphery	0.000	174
GO:0005886	plasma membrane	0.000	171
GO:0032501	multicellular organismal process	0.000	214
GO:0005549	odorant binding	0.000	25
GO:0051716	cellular response to stimulus	0.000	193

## Data Availability

The detailed data used to support the findings of this study are available from the corresponding author upon written request.
